# Genetic causal beliefs about morbidity: associations with health behaviors and health outcome beliefs about behavior changes between 1982–2002 in the Finnish population

**DOI:** 10.1186/s12889-015-1657-x

**Published:** 2015-04-17

**Authors:** Ari Haukkala, Hanna Konttinen, Nelli Hankonen, Markus Perola, Helena Kääriäinen, Veikko Salomaa

**Affiliations:** Department of Social Research, University of Helsinki, Helsinki, Finland; School of Social Sciences and Humanities, University of Tampere, Tampere, Finland; National Institute for Health and Welfare, Helsinki, Finland

**Keywords:** Genetics, Causal beliefs, Health behaviors, Attitudes, Behavior change, Obesity, Smoking, Physical activity

## Abstract

**Background:**

The role and meaning of genetic information has grown considerably in the recent decades. We examined changes in causal beliefs about morbidity as well as the associations between causal beliefs, health behaviors and obesity, and health outcome beliefs from 1982 to 2002.

**Methods:**

In five population-based risk-factor surveys (the FINRISK Studies) of individuals aged 25 to 64 years conducted from 1982 to 2002 (n = 37,503), respondents chose the most important cause of morbidity from a list of ten alternatives. Health outcome beliefs were assessed with two items. Physical inactivity and smoking status were based on self-reports and obesity was based on measured height and weight.

**Results:**

The prevalence of those who endorse genetic factors as the most important cause of morbidity increased from 4% in 1982 to 10% in 1992 and remained at that level until 2002. During the study period, lack of exercise and overweight increased, whereas inappropriate diet and stress diminished as causal beliefs about morbidity. Smokers and physically inactive were more likely to endorse genetic than behavioral causes of morbidity, whereas obese respondents were more likely to choose overweight over genetic causes of morbidity. Those who endorse genetic factors as the most important cause had more pessimistic outcome beliefs about health behavior changes, but these outcome beliefs became more positive in all causal belief groups during the study period.

**Conclusion:**

Despite increased public discussion of genomics, the relative proportion of those who endorse genetic factors as the most important cause of morbidity has remained low. However, within this group beliefs about benefits of health behavior changes have become more positive. This could indicate that increase in genomic health information does not lead to more negative appraisals of efficacy of lifestyle changes.

## Background

People hold different beliefs about the causes of health conditions. These beliefs may have a positive or negative impact on health through health-related behaviors [1]. The beliefs can be classified in various ways including social, environmental, behavioral or religious causation of diseases [2], but one important distinction has been made between genetic and behavioral/environmental causes of diseases. When people perceive that a certain condition has genetic causes, it is seen as more deterministic [3,4]. There has been a concern that increasing the significance of genetic causes of diseases leads to perceptions that other causes of diseases are less important and diseases are less preventable [5]. Furthermore, people who engage in adverse health behaviors are more likely to attribute causes of diseases to genetic rather than behavioral causes [6,7]. Although the concern that personalized genetic information will increase fatalism did not get support from a systematic review of five existing studies [8], it may still have an effect on what kind of actions are seen effective for disease prevention or treatment. It can reduce the belief in the significance of behavioral benefits to health [9] or increase the perceived effectiveness of pharmacological treatments and respectively decrease the perceived effectiveness of non-pharmacological treatments for certain diseases [10]. However, in their experimental study using vignettes, Wright et al. [11] found that genetic causal information had no effect on mild depression or obesity, but did reduce the perceived effectiveness of non-pharmacological treatments for heart disease. Genetic causal information reduced the outcome beliefs about changes in diet and exercise and reduced one’s perceived control over health problems in the case of heart disease. Although genetic information did not increase the perceived effectiveness of pharmacological treatments for heart disease, for severe depression it did [11]. It seems that genetic risk information may have different kind of outcomes depending on health problems and proposed treatments. Condit and colleagues [12] have challenged the division between behavioral and genetic causation discourse. It seems that people can hold both genetic and behavioral causal beliefs about diseases and use them in different contexts for different purposes [12,13].

Different information sources constantly influence our beliefs about the causes of various health conditions, which, as noted above, play a significant role in lay judgments of appropriate and effective responses to the management of health threats. In addition, our scientific understanding of the role of genetics has increased vastly and has been shared with the general public. In Finland, researchers have long been active in human genetics, as the relatively isolated Finnish population and a positive public opinion towards research have offered excellent opportunities to examine Finnish disease heritage [14,15] and other heritable diseases [16]. The first genetic tests were implemented in 1992 through Finnish public health care system. The analyses of Finnish newspapers [17] and TV news [18,19] from 1991 to 2000 showed that news related to human genetics have been positive and there have been less concerns about possible risks or ethical problems in human genetics compared with many other European countries [20]. According to Eurobarometer surveys from 1996 to 2010, Finland was an exception among northern European countries as there was highest support for biotechnology and high technological optimism and at the same time high engagement for biotechnology [20-22].

In this study, the first aim was to examine changes in causal beliefs about morbidity, especially changes in beliefs that genetic factors are the most important cause of morbidity, in the adult Finnish population from 1982 to 2002. Secondly, we investigated whether individuals with adverse health behaviors were more likely to attribute morbidity to genetic causes than to health behaviors. Thirdly, do those who believe that genetics are the most important cause of morbidity also hold more pessimistic outcome beliefs related to health behavior changes than do others? Finally, we tested whether all these associations have remained unchanged from 1982 to 2002.

## Methods

### Participants

The National Cardiovascular Risk Factor Survey (the FINRISK Study) has been conducted in Finland every five years since 1972. Subjects of this study are participants from five surveys from 1982 to 2002 which assessed causal beliefs about morbidity. A random sample of people aged 25–64 years from three geographical regions in 1982 and 1987, four regions in 1992, and five regions in 1997 and 2002, stratified by sex and 10-year age groups, was drawn from the Finnish population register. The total numbers of respondents from 1982 to 2002 were respectively N = 9347, N = 6479, N = 6051, N = 7158, and N = 8468. The response rates varied from 79.2% to 65.6% among men and from 85.0% to 76.2% among women in the five surveys. Participants received a mailed questionnaire that included items about sociodemographic factors, health behavior, medical history, and other health-related topics. They returned the completed questionnaires when they attended a medical examination at a health center, where their weight, height, and blood pressure were measured and a blood sample was drawn. The Ethics Committee of the National Public Health Institute approved the study protocols as did the Ethical Committee of the Hospital District of Helsinki and Uusimaa in 2002.The study sought written informed consent from participants only in 1997 and 2002.

### Measures

#### Causal beliefs about morbidity

Participants were asked, “What do you think is the most important cause for high morbidity in the Finnish adult population?” followed by a list of ten alternatives: 1) incorrect diet; 2) stress, difficult living conditions and heavy work; 3) smoking; 4) lack of exercise; 5) lack of nutrients or vitamins (soil, diet); 6) overweight; 7) heredity; 8) alcohol; 9) inadequate health care; 10) pollution, toxins etc. in the environment and in the diet. From this list, respondents were asked to select one cause. The following four items were combined into one group (“other causal beliefs”) due to small number of responses: alcohol (2.2%), pollution, toxins etc. in the environment and in the diet (4.0%), inadequate health care (0.2%), and lack of nutrients or vitamins (3.2%). As a result, the present study examined seven causal belief groups. We also created a three-category variable in order to compare the genetic causal belief group (8%) with the health behavior (61%, including incorrect diet; smoking; lack of exercise; overweight; alcohol) and social/environmental (31%, including stress, difficult living conditions and heavy work; lack of nutrients or vitamins; inadequate health care; pollution, toxins etc. in the environment and in the diet) groups. In the Results section, for brevity, we use the term “genetic causal belief group” when referring to those respondents who believe that genetics are the most important cause of morbidity.

#### Outcome beliefs about health behavior changes

Outcome beliefs were assessed with two questions: “Heart disease can be prevented by healthy lifestyles” and “Changing one’s diet in middle age is not worthwhile”. Responses to these questions are later referred to as lifestyle and diet change outcome beliefs. Response options ranged from 1 (strongly agree) to 5 (strongly disagree). Appropriate reverse calculations were made so that high values signify positive attitudes toward health behavior changes and their effectiveness in disease prevention.

#### Health behaviors and obesity status

*Current smokers* were those participants who reported smoking regularly more than once a day for at least one year and had been doing so during the preceding month. In the analyses, never smokers (52.8%) were compared with former (19.8%) and current smokers (27.4%). Body mass index (BMI) was calculated as one’s weight in kilograms divided by the square of one’s height in meters. Participants with a BMI ≥ 30 kg/m^2^ were categorized as *obese* (18.5%) and compared with non-obese participants (BMI < 30 kg/m^2^) in the analyses. Leisure time physical activity was assessed with a question where participants chose from four descriptions to depict their typical physical activity: the *physically inactive* (27.5%) were characterized with the description ‘In my leisure time I read, watch television or work in the home at tasks that do not make me move much and which do not physically exhaust me’. This group was compared with those who selected the item ‘In my spare time I walk, cycle, or exercise in some other way at least 4 h/week. This includes walking, fishing and hunting, light gardening, and so on, but excludes travel to work’ or two other items that reflected even more leisure time physical activity. The question has shown good criterion validity against morbidity and mortality [23] and moderate correlation against accelerometer counts among the working age population [24]. The sociodemographic variables were age, gender, and self-reported total years of education as an indicator of educational attainment. Education years were categorized into tertiles according to birth year separately for each study year.

### Statistical methods

First, we examined changes in the prevalence (%) of different causal beliefs between the five study years. Next, we used multinomial logistic regression models to investigate the odds of holding a health behavior causal belief and social/environmental causal belief (vs. genetic belief), with gender, age groups, educational tertiles, and study year as predictors. Dichotomous physical inactivity and obesity status variables served as dependent variables in the binary logistic regression models (adjusted for age and study year) that examined differences between seven causal belief groups, with the genetic belief group serving as a reference group. When a three-category smoking status was analyzed as a dependent variable, multinomial logistic regression was used and never smokers were compared with ex-smokers and current smokers. The interaction term between causal beliefs and study year was tested separately and the χ^2^-test for model fit improvement was utilized to determine the significance of the interaction effect. We used analysis of covariance (with age as a covariate) to examine mean differences in lifestyle and diet change outcome belief scores between causal belief groups and study years. For these analyses, we used three causal belief groups (genetic, health behavior and social/environmental). Finally, we examined whether the associations between genetic causal beliefs and outcome beliefs changed during the study period by testing the interaction term between study year and causal belief groups.

## Results

### Causal beliefs about morbidity

**Figure 1:**
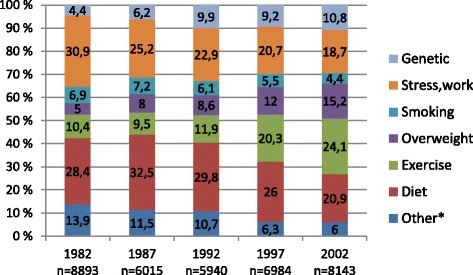
Percentages of causal beliefs about morbidity by study year among 25 to 64 -year old males and females. *Includes four items: alcohol, toxins in diet and environment, inadequate health care, lack of nutrients and vitamins.

The proportion of those who selected heredity as the main cause for high morbidity increased from 4.4% in 1982 to 10.8% in 1992 and remained at that level until 2002 (Figure 1). The proportion of those who thought that lack of exercise is the main cause of morbidity increased from 10.4% in 1982 to 24.1% in 2002. Attributions to overweight increased from 5.0% to 15.2%, while attributions to incorrect diet decreased from 28.4% to 20.9% during this same period. The percentage of those who identified stress, hard work or difficult living conditions as the main causes decreased from 30.9% to 18.7% (Figure 1). Table 1 shows that compared with the 2002 survey, respondents chose health behavior causes significantly more often than genetic causes in the earlier survey years, except in 1997. Younger age groups were more likely to choose health behavior causes than genetic causes, but we found no gender or educational differences (Table 1). Compared with the 2002 survey, respondents chose social/environmental causes significantly more likely than genetic causes in the earlier years (Table 1). Younger age groups and lower educational tertiles were more likely to choose social/environmental causes than genetic causes, but we found no gender differences (Table 1).

**Table 1:** **Multinomial logistic regression testing associations between study year and participant demographics and endorsing health behaviors or social/environmental factors as the most important cause of morbidity compared with genetic factors**

	**Genetic**	**Health behaviors**				**Social/Environmental**			
	**%**	**%**	**OR** ^**1**^	**(95% CI)**	**p**	**%**	**OR** ^**2**^	**(95% CI)**	**p**
**Study year**									
1982	4.4	53.7	1.91	1.67-2.19	<0.001	42.9	3.70	3.20-4.26	<0.001
1987	6.2	59.2	1.53	1.34-1.76	<0.001	34.5	2.20	1.91-2.54	<0.001
1992	9.9	59.1	0.97	0.86-1.09	0.599	30.9	1.37	1.21-1.55	<0.001
1997	9.2	66.0	1.17	1.05-1.31	0.005	24.8	1.24	1.09-1.40	0.001
2002 (ref.)	10.8	67.0	1.00			22.3	1.00		
**Age group**									
25-34	5.9	66.1	1.87	1.66-2.10	<0.001	28.0	1.32	1.17-1.50	<0.001
35-44	7.2	62.7	1.46	1.31-1.63	<0.001	30.1	1.17	1.04-1.31	0.008
45-54	8.9	57.6	1.09	0.98-1.21	0.111	33.4	1.06	0.95-1.18	0.315
55-64 (ref.)	9.7	56.8	1.00			33.5	1.00		
**Gender**									
Male	8.0	58.6	0.94	0.87-1.06	0.115	33.4	1.08	0.99-1.18	0.070
Female (ref.)	8.0	62.5	1.00			29.5	1.00		
**Education tertiles**									
1st	6.2	53.1	0.95	0.85-1.06	0.364	40.7	1.69	1.50-1.90	<0.001
2nd	8.3	60.9	0.97	0.87-1.07	0.836	30.8	1.41	1.27-1.55	<0.001
3^rd^ (ref.)	9.5	68.0	1.00		0.044	22.5	1.00		

^1^Model including all variables, health behavior compared with genetic causal group.

^2^Model including all variables, social/environmental compared with genetic causal group.

### Health behaviors and obesity

Table 2 shows the prevalence of obese subjects, physically inactive subjects and former and current smokers by causal belief group. Obesity and physical inactivity were predicted with logistic regression models that included age, study year and causal belief group as independent variables. Subjects in the lack of exercise causal belief group were less likely [OR = 0.82 (0.73-0.92)] to be obese than were those in the genetic belief group, but the subjects in the overweight causal belief group were more likely [OR = 1.23 (1.09-1.39)] to be obese than were those in the genetic belief group (Table 2). Physical inactivity was less likely in the lack of exercise [OR = 0.66 (0.59-0.73)] and incorrect diet [OR = 0.89 (0.81-0.98)] causal belief groups than in the genetic causal belief group (Table 2). Compared with the genetic causal belief group, being an ex-smoker was more likely in the stress and work causal belief group [OR = 1.15 (1.03-1.29)]. Current smoking was less prevalent in the diet [OR = 0.79 (0.71-0.87)], lack of exercise [OR = 0.85 (0.76-0.94)], overweight [OR = 0.74 (0.66-0.83)] and smoking [OR = 0.71 (0.62-0.81)] causal belief groups than in the genetic causal belief group, but smoking was more likely in the stress and work [OR = 1.27 (1.15-1.40)] and the other causal beliefs [OR = 1.18 (1.0-1.33)] groups compared with the genetic causal belief group. There was a significant interaction between study year and causal belief groups on smoking status (Table 2): In a more detailed examination, odds ratios between the genetic causal belief group and other groups were similar in all years when former smokers were compared with never smokers. However, when current smokers were compared with never smokers, odds ratios in 1992 differed from odds ratios in other years, as there were more smokers (31%) in the genetic causal belief group in 1992.

**Table 2:** **Percentages and odds ratios (OR) with 95% confidence intervals (CI) of obesity, physical inactivity, and ex- and current smoking by causal belief groups among males and females aged 25 to 64 years**

**Health**	**Causal belief group**							
**Behaviors**	**Genetic**	**Diet**	**Lack of exercise**	**Overweight**	**Smoking**	**Stress, work**	**Other**	**Interaction** ^**3**^
	**n = 2873**	**n = 9774**	**n = 5577**	**n = 3523**	**n = 2142**	**n = 8569**	**n = 3488**	**p-value**
Obese (%)	20.7	16.8	15.6	23.0	19.3	18.5	18.1	
OR (95% CI)^1^	1.00	0.94(0.84-1.05)	0.82(0.73-0.92)	1.21(1.09-1.39)	0.93(0.81-1.08)	0.95(0.85-1.06)	0.90(0.79-1.03)	.060
Physically inactive (%)	27.2	26.6	19.8	25.1	29.9	30.8	32.4	
OR (95% CI)^1^	1.00	0.89(0.81-0.98)	0.66(0.59-0.73)	0.90(0.81-1.01)	1.03(0.91-1.17)	1.07(0.97-1.17)	1.12(1.00-1.25)	.311
Ex-smokers (%)	19.3	19.6	20.1	20.5	22.1	19.4	19.0	
OR (95% CI)^2^	1.00	1.03(0.92-1.15)	1.05(0.93-1.199	1.01(0.89-1.15)	1.10(0.95-1.27)	1.15(1.03-1.29)	1.08(0.95-1.24)	
Current smokers (%)	27.9	24.8	26.3	23.2	21.0	32.6	31.0	
OR (95% CI)^2^	1.00	0.79 (0.71-0.87)	0.85(0.76-0.94)	0.74(0.66-0.83)	0.71(0.62-0.81)	1.27(1.15-1.40)	1.18(1.05-1.3)	.011

^1^Binary logistic regression, adjusted for age and study year.

^2^Multinomial logistic regression (never smokers as a reference), adjusted for age and study year.

^3^Interaction term between study year and causal belief groups, including age in the model.

### Health outcome beliefs

**Figure 2:**
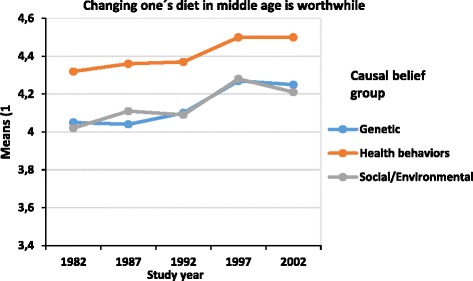
Means of diet change outcome belief by study year and causal belief groups among 25 to 64 -year old males and females. Footnotes. 1) Adjusted for age, Study year F(4,35750) = 39.5, p < 0.001, η^2^ = 0.004, Causal belief group F (2,35750) = 241.9, p < 0.001, η^2^ = 0.013, Interaction (F (8,35750) = 1.2, *p* = 0.290, *η2* = 0.0003).

**Figure 3:**
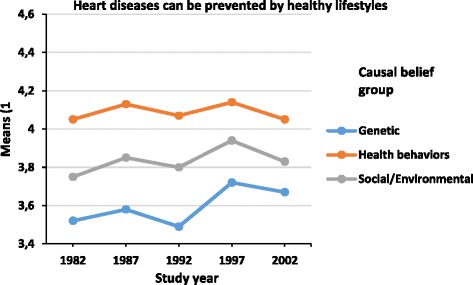
Means of lifestyle outcome belief by study year and causal belief groups among 25 to 64 -year old males and females. Footnotes. 1) Adjusted for age, Study year F (4,35783) = 22.7, p < 0.001, η^2^ = 0.003, Causal belief group F (2,35783) = 686.1, p < 0.001, η^2^ = 0.037, Interaction (F (2,35783) = 5.4, *p* < 0.001, *η2* = 0.001.

Figure 2 shows that from 1982 to 2002, diet change outcome beliefs (F (4,35750) = 39.5, *p* < 0.001, *η2* = 0.004) became more positive. Health behavior causal belief group had more positive diet change outcome beliefs compared with social/environmental and genetic causal belief groups (Table 3). There was no significant interaction between causal belief groups and study year (F (8,35750) = 1.2, *p* = 0.290, *η2* = 0.0003), indicating that the change between years was similar in all groups. Figure 3 demonstrates that also lifestyle change outcome beliefs became more positive during the study period (F (4,35783) = 23.3, *p* < 0.001, *η2* = 0.002). The genetic causal belief group held the most pessimistic beliefs, and the social/environmental causal belief group had lower means than the health behavior causal belief group (Table 3). The interaction term between study year and causal belief groups was significant (F (2,35783) = 5.4, *p* < 0.001, *η2* = 0.001), indicating that the differences between these three groups were smaller in 2002 than in 1992 and earlier.

**Table 3:** **Means and standard deviations of health outcome beliefs by causal belief groups among males and females aged 25 to 64 years**

	**Causal belief group**					
	**Genetic**	**Health behaviors**	**Social/Environ.**	**All**		
	**Mean (SD) N**	**Mean (SD) N**	**Mean (SD) N**	**Mean (SD) N**	**p** ^**1**^	**η** ^**2**^
Changing one’s diet in middle age is worthwhile	4,1	4,4	4,1	4,3		
	(1,06)	(1,01)	(1,09)	(1,06)	<0.001	0.018
	2864	21703	11119	35766		
Heart disease can be prevented by healthy lifestyles	3,6	4,1	3,8	4,0		
	(0,87)	(0,75)	(0,84)	(0,81)	<0.001	0.039
	2866	21722	11211	35799		

^1^Adjusted for age.

## Discussion

We examined how the relative importance of different causal beliefs about morbidity in the general population changed between 1982 and 2002. During that period, the proportion of those who identified physical inactivity and overweight as the main cause of morbidity increased markedly, while the proportion of those who chose inaccurate diet or stress and work-related causes diminished. This implies that the public discussion of health-related issues may affect people’s beliefs about the causes of diseases. The main interest was on those who endorse genetic factors as the most important cause of morbidity, a small yet growing proportion of the participants. In general, those who chose genetic factors as the main cause of morbidity were more likely to be obese, physically inactive and smokers than were those who chose behavioral factors as the main cause of morbidity. Although the genetic causal belief group held more negative outcome beliefs about the effectiveness of health-related lifestyle changes in preventing morbidity than the health behavior causal belief groups, these outcome beliefs became more positive from 1982 to 2002 in all causal belief groups.

The public discourse related to the prevention of obesity and public health emphasis on behavior change in general could be responsible for the fact that the combined prevalence of overweight and lack of exercise causal beliefs increased from 15% in 1982 to nearly 40% in 2002. During the same period the prevalence of obesity has increased about 10% in Finland [25]. On the contrary, the proportion of those who chose inaccurate diet decreased between 1982 and 2002. The item could be interpreted as a wrong kind of diet, which would mean different things to different people, not just too much energy in one’s diet. The causal belief item that included stress, heavy work and difficult living conditions had a large decrease despite that the public discussion on socioeconomic differences in mortality has increased. Further studies should also investigate beliefs regarding societal and psychological causation of diseases for a comparison with genetic causation beliefs [12]. Another unexpected finding was the low proportion of those who chose smoking as the most important cause of morbidity. In fact, smoking is indeed the most significant cause of both mortality and lower disability adjusted life years in high income countries [26]. However, as only a minority of people smoke regularly in Finland, people may perceive smoking as a relatively unimportant cause of morbidity at the population level. In the UK survey in which respondents were asked to list as many risk factors as they could for heart disease and cancers, smoking was the most often listed lifestyle risk factor for both groups of diseases [27]. With a similar question, smoking could have been among the most often listed causes also in the Finnish population.

In our study, the prevalence of those viewing genetics as the most important cause increased during 1982–2002 from 4% to 10%. In a UK population-based study from 2002, one third reported genetic causes for heart disease and cancers spontaneously to open-ended questions when allowed to list several causes [28]. In line with earlier studies, we found that older people were more likely to attribute morbidity to genetic causes than younger people [6,28]. However, older people have poorer genetic knowledge and health literacy than younger people [29]. In regards to the link between participants’ causal beliefs and their own health behaviors, the physically inactive and smokers were more likely to select genetic causes over the lack of exercise or inaccurate diet as a cause of morbidity. One earlier study found that the more behavioral risk factors respondents have, the more likely they endorse genetic causes [7]. Obese subjects were less likely to select lack of exercise as the main cause of morbidity, but were more likely to choose overweight than genetic causes. In a study by Wang et al. [6], those who indicated that obesity is inherited, reported engaging in less physical activity and consuming fewer fruit and vegetables. In the same study, obese subjects were more likely to indicate that obesity is inherited than were normal-weight subjects [6]. However, obvious difference to our study is that we explored causal beliefs about morbidity, not obesity.

We also found that those who selected heredity as the most important cause of morbidity more often held negative outcome beliefs about health behavior changes. Several experimental vignette studies on obesity [30], smoking [31] and heart disease [32] have shown that when a disease or health problem is depicted to have a genetic cause, people perceive changes in behavior as less helpful than medication or professional help, while some studies have not shown such consequences in perception [11,33]. In our observational study people did not receive any genetic information, but we expected that increased public discourse about genetics would increase the number of participants who endorse genetic factors as the main cause of morbidity during 1982–2002. However, we observed that outcome beliefs about benefits of lifestyle changes became more positive in this group. Sanderson et al. [28] found that people who listed genetic factors as one cause of heart disease and cancers were also more likely to specify lifestyle risk factors when allowed to list as many risk factors as possible. Our and Sanderson et al.’s [28] findings could indicate that people use “both/and” explanations more often than “either/or” explanations for diseases and that the use of different causal beliefs in different contexts is reflective as Condit and colleagues have suggested [12]. Furthermore, our result might imply that future public discussion on the advances in the genetic research does not necessarily negatively affect beliefs regarding the efficacy of lifestyle changes.

The main limitation of our study is in its assessment of causal beliefs. People were asked to indicate the main cause of high morbidity in the Finnish population, although a more common and a more specific way of assessing this would be to link the cause to a particular disease. The question has not been validated, and we do not know how people have interpreted the term “high morbidity”. A further limitation is the forced choice of only one main cause, while most people understand that there are multiple causes of morbidity in the population and that these causes create a complex network of causal mechanisms [12,34]. Because only one main cause was permitted, we do not know whether the relative significance of genetic causes increased in the population ranking of causes. Furthermore, it would have been interesting to compare different causes of diseases for one’s own health and for the population in general. Health behaviors and health outcome beliefs were self-reported and based only to single or few items. Finally, the last year when causal beliefs were assessed in the FINRISK Study was 2002. Since then positive attitudes towards biotechnology have increased in Finland as shown in Eurobarometer survey from 2010 where there was more Finnish people who have heard about biobanks and who were willing to participate those than many other European countries [22]. Furthermore, as a Dutch study [35] showed, at least some attitudes about genetic testing have changed thereafter. However, either of these studies did not explore causal beliefs or their associations with other attitudes.

This study, however, offered a rare and even unique opportunity to examine changes in causal beliefs about morbidity and associations with health behaviors and health outcome beliefs at the population level. The strengths of our study include its large and representative population-based sample, which had sufficiently high response rate, and a study protocol that has remained constant over the years. However, the number of areas covered by the study increased from three areas (eastern and western Finland) to five areas (including also the capital area and northern Finland) and in this sense the study became more representative over time. The present study provides an interesting starting point for future studies investigating the effects of new genomic findings on public beliefs and attitudes.

## Conclusions

The proportion of those who choose genetic factors as the main cause for high morbidity has increased from 1982 to 2002. During the same time period the number of those who choose lifestyle factors as the main cause has increased and beliefs about benefits of lifestyle changes have become more positive. The time frame in which the assessments were conducted overlaps with considerable public health emphasis on behavior change, which may explain the changes in behavioral attributions. Even in the societal context, which is characterized by positive attitudes and high trust towards human genetics and biotechnology, there are no clear signs that this will increase genetic determinism. It is likely that people can hold multiple causal beliefs that can be applied differently in various contexts and diseases. This does not undermine the high need for further studies to improve genomic health literacy among the population when communicating possible future genomic discoveries and treatments.
